# The Current and Potential Application of Medicinal Cannabis Products in Dentistry

**DOI:** 10.3390/dj9090106

**Published:** 2021-09-10

**Authors:** Henry Lowe, Ngeh Toyang, Blair Steele, Joseph Bryant, Wilfred Ngwa, Kaveh Nedamat

**Affiliations:** 1 Biotech R & D Institute, University of the West Indies, Mona 99999, Jamaica; lowebiotech@gmail.com (H.L.); jbryant@ihv.umaryland.edu (J.B.); 2 Vilotos Pharmaceuticals Inc., Baltimore, MD 21202, USA; ngeh.toyang@flavocure.com; 3 Flavocure Biotech Inc., Baltimore, MD 21202, USA; 4 Department of Medicine, University of Maryland Medical School, Baltimore, MD 21202, USA; 5 Brigham and Women’s Hospital, Dana-Farber Cancer Institute, Harvard Medical School, Boston, MA 02215, USA; wngwa@bwh.harvard.edu; 6 School of Medicine, Johns Hopkins University, Baltimore, MD 21218, USA; 7 Sloan School of Management, Massachusetts Institute of Technology, Cambridge, MA 02142, USA; nedamat@mit.edu; 8 Auraleaf Innovations, Toronto, ON M9B 4H6, Canada

**Keywords:** *Cannabis sativa* L., cannabinoids, periodontitis, gingivitis, dental caries

## Abstract

Oral and dental diseases are a major global burden, the most common non-communicable diseases (NCDs), and may even affect an individual’s general quality of life and health. The most prevalent dental and oral health conditions are tooth decay (otherwise referred to as dental caries/cavities), oral cancers, gingivitis, periodontitis, periodontal (gum) disease, Noma, oro-dental trauma, oral manifestations of HIV, sensitive teeth, cracked teeth, broken teeth, and congenital anomalies such as cleft lip and palate. Herbs have been utilized for hundreds of years in traditional Chinese, African and Indian medicine and even in some Western countries, for the treatment of oral and dental conditions including but not limited to dental caries, gingivitis and toothaches, dental pulpitis, halitosis (bad breath), mucositis, sore throat, oral wound infections, and periodontal abscesses. Herbs have also been used as plaque removers (chew sticks), antimicrobials, analgesics, anti-inflammatory agents, and antiseptics. *Cannabis sativa* L. in particular has been utilized in traditional Asian medicine for tooth-pain management, prevention of dental caries and reduction in gum inflammation. The distribution of cannabinoid (CB) receptors in the mouth suggest that the endocannabinoid system may be a target for the treatment of oral and dental diseases. Most recently, interest has been geared toward the use of Cannabidiol (CBD), one of several secondary metabolites produced by *C. sativa* L. CBD is a known anti-inflammatory, analgesic, anxiolytic, anti-microbial and anti-cancer agent, and as a result, may have therapeutic potential against conditions such burning mouth syndrome, dental anxiety, gingivitis, and possible oral cancer. Other major secondary metabolites of *C. sativa* L. such as terpenes and flavonoids also share anti-inflammatory, analgesic, anxiolytic and anti-microbial properties and may also have dental and oral applications. This review will investigate the potential of secondary metabolites of *C. sativa* L. in the treatment of dental and oral diseases.

## 1. Introduction

Oral and dental diseases contribute to a significant economic burden of productivity loss, particularly in low- and middle-income countries that lack the appropriate resources to treat such conditions. Regions such as Western Europe, Australasia, Central Europe, High-Income North America, and High-Income Asia Pacific were found to have the highest levels of per capita dental expenditures, with untreated dental caries (tooth decay) being the most prevalent health condition [1]. On the same tangent, oral cancers have a very high mortality rate in Jamaica [2].

In 2010, the global economic impact of dental diseases was reported to be an estimated USD 442 billion [3]. In 2015, the estimated direct and indirect costs of dental diseases totalled to USD 544.41 billion [1]. Regions with the highest levels of per capita dental expenditures included North America, Australasia, Western Europe, Asia Pacific and East Asia [1]. 

Additionally, in 2015, severe tooth loss was estimated to account for 67% of global productivity loss due to dental diseases [1]. Severe periodontitis (21%) and untreated caries (12%) followed [1]. In 2017, the global economic impact of productivity loss due to periodontitis was estimated to be EUR 44.28 B (USD 54 B) and EUR 20.50 B (USD 25 B) in direct and indirect costs, respectively [4]. In 2018, the economic impact of productivity loss due to periodontal disease in the Europe and the U.S.A. was estimated at EUR 149.52 B and EUR 122.65 B, respectively [5], with oral diseases affecting an estimated 3.5 billion people [6].

Table 1 is a list of the most common dental and oral diseases.

### 1.1. History of Herbal Remedies to Treat Oral and Dental Diseases

Medicinal plants such as *Acacia catechu* (L.f.) Willd., *Spilanthes* spp., *Wrightia tinctoria* R.Br., *Cannabis sativa* L., *Ophiopogonis radix*, *Salvia officinalis* L., *Syzygium aromaticum* (L.) Merr. and L.M.Perry (clove), *Allium sativum* L. (garlic), and *Datura stramonium* L. have been utilized across traditional Asian, African and Indian medicine for several hundred, and possibly thousand years, to treat many ailments. These include oral and dental diseases such as oral ulcers, periodontal abscesses, oral mucositis, oral microbial infections, oral inflammatory diseases, toothache, pyorrhea, acute dental pulpitis, halitosis and sore throat [11,12,13,14,15,16,17,18,19,20,21,22]. More recently, traditional Chinese medicine (TCM) has been utilized in the treatment of oral diseases including, but not limited to oral lichen planus, recurrent aphthous stomatitis, oral leukoplakia, and Sjögren’s syndrome [11].

In addition to utilization in other traditional medicinal systems, medicinal plants such as *Mentha piperita* L. (peppermint), *Melaleuca alternifolia* (Maiden and Betche) Cheel (tea tree oil), *Calendula officinalis* L., *Aloe vera* L., *Citrus limon* (L.) Osbeck, *Camomilla matriciana, Rosmarinus officinalis* L. (rosemary), *Thymus vulgaris* L. (thyme), and Eugenol (a compound produced by multiple medicinal plants), are also widely utilized in western complementary medicine in the treatment of oral and dental diseases [23,24,25,26].

### 1.2. History of Cannabis sativa L. in the Treatment of Oral and Dental Diseases

*Cannabis sativa* L. has a long history in traditional Asian, African and Indian medical systems/pharmacopoeias, for the treatment of oral and dental diseases since at least 2700BC in China [27,28]. As it relates to the treatment of oral and dental disease, in these traditional medical systems *C. sativa* L., was utilized for toothache management, though it is also likely that the plant may have also been used in the treatment and prevention of dental caries and reduction in gum inflammation [29].

*C. sativa* L. produces many pharmacologically active secondary metabolites including cannabinoids, terpenes and flavonoids that share anti-inflammatory, antioxidant anti-microbial, analgesic, anti-cancer, anxiolytic properties [30,31,32,33,34,35,36,37,38,39]. This, along with the finding that cannabinoid receptors are also distributed within the oral cavity has resulted in an increased focus toward alternative, cannabinoid-based pharmaceutical compositions for the maintenance of oral health and in the treatment of oral diseases. 

There is also an increasing shift toward alternative natural oral hygiene products. One major reason is the increasing resistance to synthetical antimicrobials and the possible adverse effects of chemical agents [40]. To date, cannabinoid-based pharmaceutical compositions have been patented for the maintenance of general oral hygiene and for specific oral and dental diseases. Table 2 below lists examples of such patents. 

This review will attempt to make an argument for the therapeutic potential of secondary metabolites of *C. sativa* L. against oral and dental diseases, based on the aforementioned therapeutic properties of metabolites and the implication of the ECS in oral and dental diseases. However, further studies will be required to elucidate mechanisms of action, efficacies, safeties and toxicities of these secondary metabolites.

## 2. Current Uses of Cannabinoids in Modern Dentistry

There is currently a wide range of cannabinoid-based oral products currently on the market, and studies being conducted on cannabinoid-based pharmaceuticals in general, are increasingly positive and evidence-based. These products include cannabidiol (CBD) capsules, CBD pills, CBD Hemp Oil Tinctures, CBD Oil, CBD-infused toothpastes, CBD Oral Sprays, CBD-infused mouthwashes, CBD chewing gum and even CBD-infused dental fillings [40,41,42,43]. The aforementioned products are primarily used as analgesics to provide relief from tooth pain and gum soreness, and as anti-microbials and anti-septic agents to maintain oral hygiene, and anti-inflammatory agents to control inflammation of the gums [44,45]. Despite the myriad of existing cannabinoid-based oral products, the scientific literature on the safety, efficacy, toxicity and quality of these pharmaceuticals, is limited. Ultimately, the regulatory framework governing these compounds and their use thereof, as defined by the U.S. Food and Drug Administration, is also in its nascent stage of development. On this tangent, it is suggested that patients seek professional medical advice before using cannabinoid-based oral products. These regulatory frameworks are built on rigorous, scientific- and evidence-based data, which are currently lacking. 

In one preliminary observation, scientists found that cannabinoids were more effective in reducing bacterial colony count in dental plaques when compared to Colgate and Oral B, industry-standard, synthetic oral hygiene products [46]. Due to antimicrobial resistance, it was then concluded that these cannabinoids may be a safer alternative to traditional synthetic oral hygiene products [46]. In another study, the efficacy of two mouthwash products, one containing a <1% cannabidiol (CBD) per weight and another containing < 1% cannabigerol (CBG) per weight, respectively, were investigated against total-culturable bacteria from dental plaque samples [47]. In comparison to chlorhexidine 0.2%, frequently used in traditional synthetic mouthwash, both cannabinoid-infused mouthwash products demonstrated very similar bactericidal efficacy [47].

**Table 2:** Examples of cannabinoid-based pharmaceutical compositions have been patented for the maintenance of general oral hygiene and for specific oral and dental diseases.

	Utility	Reference (Patent Number)
1.	Cannabis-based composition comprising cannabis-extract, derivatives, and/or at least one synthetic cannabinoid intended for the treatment of dental pulp infection, pulp inflammation, dental (jaw) bone defects	[48]
2.	Cannabinoid-based oral care composition (tooth paste, a tooth powder, or a mouthwash solution) for the treatment of oral infectious disease, including periimplantitis, periodontitis, oral mucositis, and dental pain. Cannabinoid may be cannabidiol and/or cannabigerol.	[49]
3.	Extract of C. *sativa* L. (toothpaste, oral cleanser, or oral spray) for the treatment of dental caries.	[50]
4.	Cannabinoid-based chewing gum compositions intended for the alleviation of pain.	[51]

### 2.1. Targeting the Endocannabinoid System (ECS)

The Endocannabinoid System (ECS) is a network of proteins (receptors, their ligands and biosynthesis and degradative enzymes) widely distributed throughout mammalian tissues and cells in virtually animal species [52,53]. The ECS is primarily responsible for maintaining internal homeostasis and directly influences physiological process regulating anxiety, feeding behavior/appetite emotional behavior, depression, nervous functions, endocrine regulation, energy balance, neurogenesis, neuroprotection, reward, cognition, learning, memory, pain, sensation, fertility, pregnancy, and pre-and post-natal development [54,55,56,57,58,59,60,61,62]. The ECS plays a role in multiple physiological and pathophysiological processes, making it a valuable target for the treatment of many diseases and disorders such as multiple types of cancers, cardiovascular diseases, neurodegenerative diseases, psychiatric disorders, mood and anxiety disorders, obesity, and substance-abuse disorders [63,64,65,66,67,68,69,70,71,72,73,74,75]. On the same tangent, it is also suggested that Squamous Cell Carcinoma of the Oral Tongue (SCCOT) may be the result of a dysregulation of endocannabinoid system signaling [76].

Phytocannabinoids, referred to as classical cannabinoids, are a class of terpenophenolic compounds that are naturally produced by *C. sativa* L. plant [77,78]. Over 100 cannabinoids have been isolated from *C. sativa* L. to date [79]. The two major, most studied and most abundantly produced phytocannabinoids are Δ^9^-THC (Δ^9^-Tetrahydrocannabinol) and Cannabidiol (CBD). Others include Tetrahydrocannabivarin (THCV), Cannabigerovarin (CBGV), Cannabichromene (CBC), Cannabichromevarin (CBCV), Cannabigerol (CBG), Cannabivarin (CBDV), Cannabivarin (CBV), and Cannabicyclol (CBL). The chemical structures of major secondary metabolites produced by *C. sativa* L. are shown in Table 3 below. 

Due to the potent properties of the major cannabinoids, they may have therapeutic potential against oral and dental diseases. Other major secondary metabolites produced by *C. sativa* L. that have a wide range of significant of therapeutic benefits are flavonoids and terpenes. Secondary metabolites are organic compounds produced by microorganisms and plants that play roles in a number of processes that give the plant or microorganism comparative advantage, may play a role in survival of the microorganism or plant, play a role in the aroma (terpenes), pigmentation and flavour of the plant (flavonoids), and that may have pharmacological/health benefits [80,81,82,83]. In addition to these roles, many cannabinoid- and non-cannabinoid secondary metabolites produced by *C. sativa* L., are analgesic, antimicrobial, anti-cancer, anti-inflammatory, anxiolytic, anti-depressant, relaxant, and antioxidant [35,39,84,85,86,87,88,89,90,91,92,93,94].

Together these cannabinoids and non-cannabinoid molecules work synergistically to enhance the biological effect of *C. sativa* L. This is known as the “Entourage Effect” [95,96,97]. As such, pharmaceuticals made of these secondary metabolites may have a role in the treatment of oral and dental diseases. Table 3 lists major secondary metabolites of *C. sativa* L. and their properties that could make them useful in dentistry.

**Table 3:** Chemical structures of the major secondary metabolites (cannabinoids, terpenes and flavonoids) of *Cannabis sativa* L. with potent properties that may make them useful in the treatment of oral and dental diseases.

	Chemical Structures of Major Secondary Metabolites of *Cannabis sativa* L.	Significant Properties	References
**Major cannabinoids**			
1.	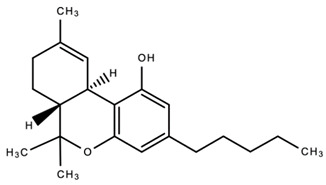 Delta 9-tetrahydrocannabinol (Δ9-THC)	Anti-microbial Anti-inflammatory Analgesic Antioxidant Anti-cancer Anti-tumor	[84,98,99,100,101,102,103,104,105]
2.	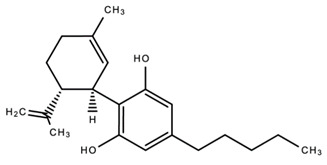 Cannabidiol (CBD)	Anti-microbial Anti-inflammatory Analgesic Anti-cancer Anti-metastatic Antioxidant Analgesic Anti-nociceptive	[35,84,98,99,100,101,102,103,106,107,108,109,110]
3.	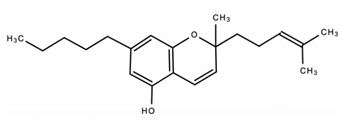 Cannabichromene (CBC)	Anti-microbial Antibacterial and Anti-fungal Analgesic Anti-nociceptive Antioxidant Anti-inflammatory Anti-depressant	[84,111,112,113,114,115,116]
4.	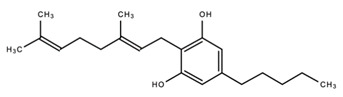 Cannabigerol (CBG)	Anti-microbial Analgesic Antioxidant	[84,117,118,119]
5.	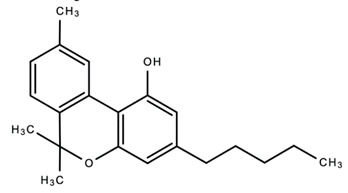 Cannabinol (CBN)	Anti-microbial Analgesic Antioxidant	[84,120,121]
**Major Terpenes**			
1.	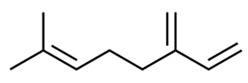 β-myrcene	Antimicrobial Antioxidant Potent analgesic Antioxidant; neuroprotective; anti-inflammatory Anti-cancer.	[85,86,122,123,124,125]
2.	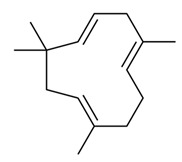 α-humulene	Antimicrobial Antioxidant Anticancer Anti-inflammatory Analgesic Angiogenic	[39,86,122,124,125,126,127,128,129,130]
3.	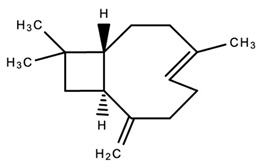 β-caryophyllene	Antimicrobial Antioxidant Anti-Cancer Anti-inflammatory Analgesic Anxiolytic	[86,122,125,130,131,132,133,134]
4.	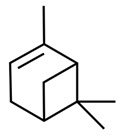 (+)-α-pinene	Antimicrobial Anti-inflammatory Antioxidant Analgesic	[92,122,123,125]
5.	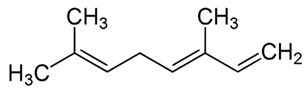 trans-β-ocimene	Antifungal; antibacterial; antioxidant; antiviral; anti-inflammatory	[87,88,89,90,91,122,125]
6.	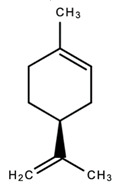 (-)-limonene	Antimicrobial Antioxidant Analgesic Anti-inflammatory; antioxidant; antiviral; antidiabetic; anticancer Antidepressant; Anticonvulsant Anti-cancer Anxiolytic	[35,86,122,125,135,136,137,138,139,140]
7.	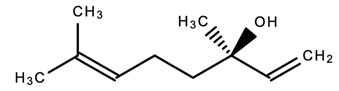 Linalool (Lavender scent)	Anxiolytic; anti-inflammatory; antimicrobial; anticancer; antidepressant Antioxidant	[39,85,122,125]
8.	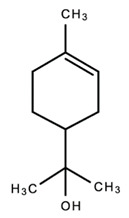 α-terpineol	Antimicrobial; Anti-inflammatory; Analgesic; Nociception inhibition; Antimicrobial	[35,92,122,125,141,142]
**Major Flavonoids**			
1.	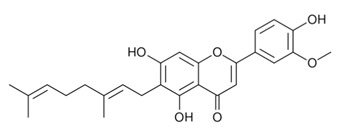 Cannflavin A	Analgesic Anti-inflammatory	[33,143,144,145,146]
2.	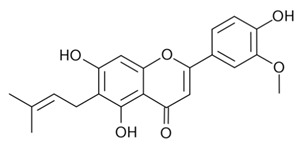 Cannflavin B	Analgesic Anti-inflammatory	[33,143,144,145,146]

Endogenous cannabinoids such as Anandamide (AEA/N-arachidonoylethanolamine) and Palmitylethanolamide (PEA) have also demonstrated analgesic effects [147]. Synthetic cannabinoids such as HU-120, HU-308, HU-320, WIN-55,212-2, JWH-133, JWH-015, nabilone, also demonstrate analgesic, anti-microbial, anti-inflammatory, anti-cancer, anti-angiogenic, anti-metastatic properties and may also be useful in the treatment of oral and dental diseases [148,149,150,151,152,153,154,155].

### 2.2. Implications of Cannabinoid Receptors in the Mouth

Cannabinoid receptors such as the transient receptor potential vanilloid channel type 1 (TRPV_1_), cannabinoid receptors type 1(CB_1_) and cannabinoid receptors type 2 (CB_2_) in addition to other cannabinoid receptors located in the salivary glands may be novel therapeutic targets in the treatment of certain oral diseases [156,157,158]. Cannabinoid receptors located in salivary glands (particularly submandibular acinar cells) have also been implicated in the regulation of salivation and saliva content and thus may even be useful in the treatment of Xerostomia (“dry-mouth”) [158,159]. On this same tangent, AM251 and AM630, CB1 and CB2 antagonists, respectively, were shown to block the inhibitory effects of anandamide (AEA) on saliva secretion, and in doing so, increased saliva secretion [158].

### 2.3. Potential Applications of CBD and Other Secondary Metabolites in Modern Dentistry

#### 2.3.1. Emerging Trends and Potential Value of Medical Cannabis in Dentistry

Now than ever before, there is an increasing acceptance of the use of medical cannabis in Dentistry. Dentists are increasingly exploring and inventing more innovative, natural, safer, and more efficient, but less expensive alternatives to traditional synthetic oral medications. Despite this increasing interest, more rigorous scientific studies are required on cannabinoid-based oral products. The financial potential for cannabis-based oral hygiene products is also very promising, especially with the growth in demand and innovation in the field.

#### 2.3.2. Toothache

Toothaches are a global, public health crisis and are one of the most common, if not the most common dental disease and the most common cause of oral pain [160,161]. Toothaches may typically be caused by an irritation, infection, or injury to the tooth, hypersensitivity of the nerves, damage to surrounding structures of the tooth, or decay of the tooth.

Although studies are limited and mechanisms of action not yet elucidated, the analgesic properties of cannabinoids such as Delta 9-tetrahydrocannabinol (Δ^9^-THC), Cannabidiol (CBD) and Cannabigerol (CBG) have implicated them in the treatment of toothaches [41,162,163]. The analgesic properties of the cannabinoids may also make them useful in pain-management for tooth extractions and post-operative pain management.

#### 2.3.3. Burning Mouth Syndrome

Burning Mouth Syndrome is a neuropathic pain condition of the tongue, lips, gum, palate and/or other areas of the oral cavity, which is characterized by chronic or recurrent burning sensation [164,165]. Although the causes have not been fully elucidated, studies suggest that the condition may be due to dysregulation of the taste and sensory nerves of the peripheral or central nervous system [166,167,168]. Multiple studies have investigated and confirmed the safety and efficacy of cannabinoids, in some form or another, against the symptoms of Burning Mouth Syndrome [169].

Components of the ECS have been implicated in the pathogenesis of Burning Mouth Syndrome [156,170]. A 2014 study by Borsani and colleagues identified an increase in the expression of transient receptor potential vanilloid channel type 1 (TRPV_1_) and cannabinoid receptors type 2 (CB_2_), but a decrease in expression of cannabinoid receptors type 1 (CB_1_) in epithelial cells of the tongue [156,170]. These studies suggest that the endocannabinoid system may be a potential target for the treatment of Burning Mouth Syndrome.

#### 2.3.4. Dental Caries

Dental caries, more simply referred to as “cavities” or “tooth decay”, is a very common condition that affects approximately 2.3 billion people worldwide, including 530 million children [6]. Dental caries is the result of several factors including plaque- and biofilm-forming bacteria such as *Streptococcus mutans* or *Lactobacillus* spp., frequent intake of sugary foods that cause acid build-up, lack of adequate teeth cleaning and subsequent demineralization/erosion of the enamel.

Due to antibacterial properties, cannabidiol may have therapeutic applicability in the treatment of dental caries [47]. Other major cannabinoids such as cannabichromene, cannabigerol, Delta (9)-tetrahydrocannabinol, and cannabinol also demonstrated potent antibacterial activity against a variety of clinically relevant methicillin-resistant Staphylococcus aureus (MRSA) strains and so, may also have anti-bacterial activity against bacteria implicated in oral and dental diseases [84,94,117,118]. Cannabinoid-infused mouthwashes have also demonstrated inhibition of bacterial activity in dental plaque samples with the same efficacy as chlorhexidine a common disinfectant and antiseptic agent [47]. Cannabidiol-supplemented tooth polishing powder has also been reported to inhibit dental plaque bacteria [47]. Numerous sources of anecdotal evidence strongly supports the use of a few drops of CBD oil per day for its antibacterial potential against said bacteria.

#### 2.3.5. Dental Anxiety

Dental anxiety is a legitimate fear of anticipated pain and subsequent avoidance of professional dental care [171]. It is a phenomenon that generally begins in childhood due to conditioning from fearful parents [172,173], and is prevalent globally. Culture and norms also influence an individual’s conditioning [174]. As a result, an individual’s oral health will deteriorate to a point where even their general health, sleep pattern, overall quality of life, self-esteem, social interactions with others, and professional and personal relationships may be significantly affected [174,175]. Multiple studies even show a positive correlation between dental anxiety and the development of dental diseases [172,176,177,178]. Dental anxiety is a frequent and significant issue for dentists globally and has even been shown to be a contributing factor to their own stress levels [171,179].

Strategies that have been developed to combat/manage dental anxiety include non-pharmacological approaches such as (1) establishment of good communication and rapport between dentist and patient, (2) systemic desensitization (3) hypnosis (4) cognitive behaviour therapy, a type of psychological therapy aimed at removing the negative thoughts associated with dental anxiety and phobia, and (5) pharmacological interventions such as intravenous sedation, inhalation sedation, local and general anaesthesia [179,180,181,182,183,184,185].

Most recently, the therapeutic potential of cannabidiol for the treatment of dental anxiety has also been investigated due to its anxiolytic, panicolytic and anti-compulsive properties. One report suggests that 15–30 milligrams of CBD applied sublingually before a dental appointment may be efficacious against dental anxiety and dental pain [186,187].

#### 2.3.6. Periodontal Disease

Periodontal disease is an inflammatory gum disease caused by bacterial plaque build-up, and characterized by inflammation and even bleeding of the gum. It may be considered the intermediate stage in the general progression of gum disease. Periodontal diseases include gingivitis (early-stage gum disease) and periodontitis (severe, advanced-stage gum disease), both of which are characterized by irritation and inflammation of the gum. If left untreated, the disease leads to progressive alveolar bone loss and eventually, tooth loss. Furthermore, these effects are not solely localized, but manifest systemically.

The endocannabinoid system may be a promising target in the treatment of periodontal disease as it has been shown to play a role in the modulation/suppression of inflammatory responses by periodontal ligament (PDL) cells [188,189]. The endocannabinoid anandamide (AEA) appears to play a modulatory role in periodontal inflammation and a role in the immunosuppression of human periodontal ligament cells’ (hPdLCs’) host response to *Porphyromonas gingivalis lipopolysaccharide (P. gingivalis LPS)* [188,189]. On the same tangent, in human patients with periodontitis, AEA expression is upregulated in gingival crevicular fluid after periodontal surgery [190]. AEA has also been shown to preservice the cellular integrity of hPdLCs [189]. Palmitoylethanolamide (PEA) also exacerbated the proinflammatory effects of AEA [189]. Cannabinoids such as THC and CBD, CBC, and CBG, demonstrate potent anti-inflammatory and analgesic effects that may make them very useful in the treatment of inflammation-based gum diseases [84,105,110,111,113,115,188,189,190,191,192].

Associated co-morbidities on the basis of epidemiological, clinical intervention and animal model-based studies include but are not limited to cardiovascular disease, diabetes, Alzheimer’s disease, rheumatoid arthritis, cancer, adverse pregnancy outcomes, inflammatory bowel disease and respiratory disease [193].

Traditionally, much of the focus of the treatment of periodontal disease has been on reducing the bacterial load. However, an opportunity exists to modulate the host inflammatory response as well, making use of the anti-inflammatory properties of cannabinoids. Those suffering from severe periodontitis exhibit elevated levels of pro-inflammatory mediators and neutrophils in their blood and local periodontal treatment reduces these inflammatory markers systemically [194]. Of particular interest is the potential for the existence of synergistic ratios between cannabinoids which when combined, can produce optimal anti-bacterial, anti-inflammatory, antioxidant and analgesic effects. One example is between CBD and CBG where studies on neuroinflammation, a key factor in amyotrophic lateral sclerosis (ALS) show that, when combined, their benefits are enhanced [194]. More research needs to be conducted in the field of oral health to see if synergies exist for this application.

With the recent COVID-19 pandemic, researchers have been exploring the role that cytokine storms play in the viral infection and in particular, the role of interleukin-6 (IL-6) [195]. Studies suggest that a predictor of COVID-19 pulmonary complications is elevated levels of IL-6. Periodontitis increase levels of IL-6 both locally and systemically meaning treatment and prevention of periodontal disease can lower levels of IL-6 and, therefore, improve respiratory outcomes of COVID-19 infection, reducing mortality [195]. In a case–control study, periodontitis was found to be associated with COVID-19 complications including death, ICU admission, and need for assisted ventilation [196]. Systemically, those COVID-19 patients with periodontitis showed significantly higher blood levels of white blood cells, D-dimer and C Reactive Protein [196]. These biomarkers are linked to worse disease outcomes. 

#### 2.3.7. Oral Mucositis and Other Forms of Oral Cancers

Oral mucositis is a disease characterized by inflammation and ulceration of the mucous membranes lining the gastrointestinal tract. Oral mucositis is regarded as the most common debilitating complication of cancer-related chemotherapy and radiation that kills both malignant and healthy tissues [197]. Though evidence is limited, the antioxidant properties of cannabidiol suggest that it may be used to control the oxidative stress associated with oral mucositis [198].

Cannabinoid CB_1_ and CB_2_ receptor are up-regulated in certain cancer types and thus, may be potential targets to exploit in the treatment of cancer and other diseases [199]. Multiple cannabinoids have demonstrated antioxidant, anti-metastatic, anti-tumorigenic, apoptotic and anti-angiogenic properties in various cancer cell lines and thus may have therapeutic benefits in treating oral cancers [104,157,200,201,202,203,204,205,206,207,208,209]. Table 4 lists particular potential applications of secondary metabolites against oral and dental diseases.

## 3. Conclusions and Future Direction

Despite the vast anecdotal evidence of the use of cannabis and cannabinoids to treat oral and dental disorders, there is limited rigorous scientific evidence for the use of cannabinoids in dentistry. It should be noted, however, that there is generally strong evidence to support the wide therapeutic window and properties of cannabinoids, including, but not limited to their analgesic, antioxidant, anti-inflammatory, anti-microbial, anti-pruritic and anti-cancer properties. For these reasons, cannabinoids may have significant applicability in dentistry for the treatment of (1) toothaches, (2) bacterial infections causing periodontitis, gingivitis, periodontal disease, dental caries, salivary gland infections and abscesses, (3) inflammation-based oral diseases (4) oral and salivary gland cancers (5) Burning Mouth Syndrome, (6) dental anxiety, and (7) for general maintenance of oral hygiene. In addition to their wide therapeutic window, cannabinoids may also be a safer alternative to conventional, synthetic drugs used to treat oral and dental diseases, and commercial oral and dental products, in general.

In order for cannabinoids and cannabinoid-based products to become mainstay in conventional dentistry, further rigorous scientific studies are required to confirm their safety, tolerability, toxicity, efficacy, optimal dosages and optimal delivery systems in the treatment of oral and dental diseases. It is only until then that we will begin to see the regulatory framework governing these compounds and their use thereof, as defined by the U.S. Food and Drug Administration, become more accepting. This includes further studies into elucidating the specific mechanisms of action of secondary metabolites of *C. sativa* L. in various oral and dental diseases, and in the case of dental caries, elucidating the minimum inhibitory concentration (MIC), minimum bactericidal concentration (MBC), and the half maximal inhibitory concentration (IC_50_) values of each cannabinoid-based pharmaceutical against causative agents of dental caries.

Patients should seek professional medical advice before using cannabinoid-based oral products.

## Figures and Tables

**Table 1:** The most common oral and dental diseases and the global prevalence of each.

	Dental and Oral Diseases	Prevalence/Incidence	Reference
1.	Dental Caries/Cavities (Tooth Decay)		
	i Dental caries of permanent teeth ii Dental caries of primary teeth in children	2.3 billion 530 million	[6]
2.	Gum (Periodontal) Disease including gingivitis	10% of global population	[7]
3.	Periodontitis (severe periodontal disease)	20–50% of global population	[8,9]
4.	Oral Cancer (that is, cancers of the lip, oral cavity, and oropharynx)	657,000 new cases annually	[6]
5.	Oro-dental trauma	Approximately 1 billion people have had traumatic dental injuries (TDIs) at some point in their lives.	[10]

**Table 4:** Potential Applications of secondary metabolites of *C. sativa* L. in dentistry.

	Potential Applications of Secondary Metabolites of *C. sativa* L. in Dentistry	Appropriate Property of Secondary Metabolite	Reference
	**Cannabinoids**		
1.	General oral hygiene (Cannabidiol, delta9-tetrahydrocannabinol ajulemic acid, Cannabigerol)	Antifungal Antibacterial	[41,84,93,105,117,118,210]
2.	Toothache (Cannabidiol, HU-320)	Analgesic	[41,151,163]
3.	Dental caries/cavities (Cannabidiol, Cannabigerol and Delta9-tetrahydrocannabinol)	Anti-bacterial Analgesic	[41,47,48,84,93,117,118,191,211]
4.	Abscesses (Cannabidiol and delta9-tetrahydrocannabinol)	Anti-bacterial Anti-pruritic	[191]
5.	Prevention of biofilm attachment on teeth (Cannabidiol and delta9-tetrahydrocannabinol)	Anti-bacterial	[84,191]
6.	Burning Mouth Syndrome (Cannabidiol)	Analgesic	[191]
7.	Oral and Salivary Gland Cancers (Cannabidiol)	Anti-cancer Anti-metastatic	[191]
8.	Periodontitis (most severe form of gum disease) (Cannabidiol, HU-320, delta9-tetrahydrocannabinol, AEA)	Anti-bacterial Anti-inflammatory Analgesic	[84,151,188,191,212,213]
9.	Periodontal (Gum) disease (Cannabidiol, delta9-tetrahydrocannabinol, Cannabigerol and HU-320)	Anti-bacterial Anti-inflammatory Analgesic	[84,93,117,118,151,211]
10.	Gingivitis (Cannabidiol, delta9-tetrahydrocannabinol, Cannabigerol, and HU-320)	Anti-bacterial Anti-inflammatory Analgesic	[84,93,117,118,151,211]
11.	Oral Mucositis and other forms of oral cancer (Cannabidiol, delta9-tetrahydrocannabinol, JWH-133m, WIN-55,212-2, Cannabinol, Cannabicyclol)	Anti-bacterial Anti-cancer Anti-metastatic Anti-inflammatory Analgesic Antioxidant	[84,154,191,192,198,214]
12.	Dental Anxiety (Cannabidiol)	Anxiolytic	[191,215]
13.	Sleep issues resulting from dental anxiety (Cannabidiol and delta-9-tetrahydrocannabinol (THC))	Relaxant	[216]
14.	Indirect enamel protectant (Cannabidiol and delta-9-tetrahydrocannabinol (THC))	The anti-bacterial properties of CBD and THC could indirectly protect the enamel by prevent plaque build-up that could ultimately lead to erosion of the enamel.	[84,191]
15.	Remineralization of enamel (Hemp oil)		[211]
16.	Improvement of tooth sensitivity (Hemp seed oil, Cannabigerol, and CBD oil)		[211,216,217,218]
17.	Stimulation of jaw bone osteogenesis/regeneration (Cannabidiol and delta-9-tetrahydrocannabinol (THC))	Stimulates osteogenesis in bone fracture healing	[219,220,221,222]
18.	Decrease in bone resorption in experimental periodontitis in rats (Cannabidiol)	Anti-inflammatory Decreases alveolar bone loss (in rat model)	[223]
19.	Salivary gland bacterial infection (Cannabidiol and delta9-tetrahydrocannabinol, cannabigerol)	Anti-bacterial Anti-inflammatory Analgesic	[93,117,118,191,224,225]
20.	Digestive issues associated with anesthesia and numbing agents (Cannabidiol)	Anti-emetic Anti-nauseant	[216]
21.	Temporomandibular Joint (TMJ) Disorder (Cannabidiol)	Analgesic	[226]
22.	Osseointegration of dental implants (HU-308—a CB2-specific agonist)	Stimulation of osteoblastic bone formation and inhibition of osteoclastic bone resorption via activation of CB2 receptors in osteoblasts and osteoclast, and subsequent maintenance of bone mass.	[227]
	**Flavonoids**		
1.	Toothaches, (Cannflavins A and B)	30× more analgesic than aspirin Anti-inflammatory	[33,143,144,228]
2.	Oral cancers that are characterized by increased production of reactive oxygen species. (Flavonols (e.g., quercetin and kaempferol))	Antioxidant;	[229]
3.	Inflammation-based oral diseases such. Oral cancers that are characterized by increased production of reactive oxygen species. (Flavanones)	Antioxidant; anticancer; anti-inflammatory	[229]
4.	Inflammation-based oral diseases. Oral cancers that are characterized by increased production of reactive oxygen species. (Anthocyanins)	Antioxidant and anti-inflammatory	[229]
	**Terpenes**		
1.	Toothache and other oral disorders that cause pain (β- caryophyllene, α-terpineol, Myrcene)	Analgesic	[35,85,86,92,141,142]
2.	Dental Anxiety (E.g. Linalool)	Anxiolytic	[86]
3.	Inflammation-based oral diseases such as gingivitis, periodontal disease and periodontitis. α-terpineol(Linalool, Myrcene, α-Pinene, Ocimene, β- caryophyllene, Limonene)	Anti-inflammatory	[85,92]
4.	Oral cancers that are characterized by increased production of reactive oxygen species. (Myrcene, Limonene, Linalool, α-terpineol, α-Humulene, Ocimene)	Antioxidant Anticancer	[39,86,87,88,89,90,91]
5.	Oral diseases such as gingivitis, periodontal disease, periodontitis and salivary gland infections that are characterized by bacterial-plaque build-up and bacterial infections. (Ocimene, α-terpineol, Linalool, α-Pinene, Limonene)	Anti-microbial (anti-bacterial)	[35,86,87,88,89,90,91,92,141,142] (Cavaleiro et al., 2015)

## Data Availability

Data sharing is not applicable to this article. No new data were created or analyzed in this study.

## Sample Availability

Samples of the compounds are not available from the authors.
